
JOURNAL INFORMATION
==============================
NLM Title Abbreviation: Anim Front
Journal ID: Anim Front
NLM Journal ID: 101754918
Journal ID: anfron

Animal Frontiers: The Review Magazine of Animal Agriculture

ISSN: 2160-6056
EISSN: 2160-6064
Publisher: Oxford University Press

ARTICLE INFORMATION
==============================
PMCID: PMC11700614
DOI: 10.1093/af/vfae046
Article ID: vfae046
Article version: 1
Subjects: Society News, AcademicSubjects/SCI00960

News

Publication date: 2024 Dec
Electronic publication date: 2025 Jan 4
Volume: 14
Issue: 6
First page: 76
Last page: 82
Copyright: Copyright © 2024 American Society of Animal Science.
Copyright year: 2024
License: This is an Open Access article distributed under the terms of the Creative Commons Attribution License (https://creativecommons.org/licenses/by/4.0/), which permits unrestricted reuse, distribution, and reproduction in any medium, provided the original work is properly cited.
License URL: https://creativecommons.org/licenses/by/4.0/

Page count: 7

==============================

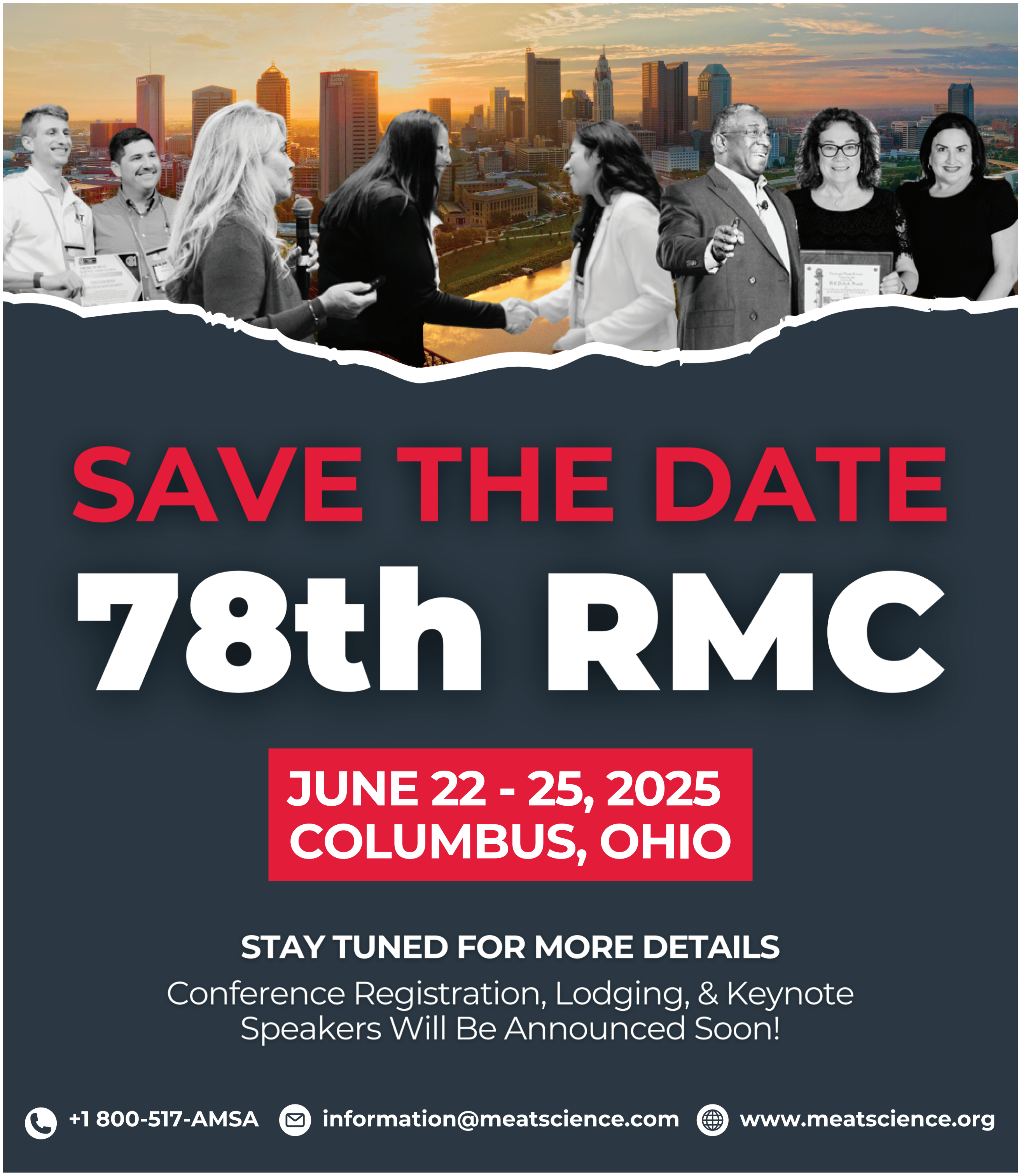

American Meat Science Association

RMC 2025 RECIPROCAL MEAT CONFERENCE

THE OHIO STATE UNIVERSITY
COLLEGE OF FOOD, AGRICULTURAL, AND ENVIRONMENTAL SCIENCES

https://doi.org/10.1093/af/vfae046

AMERICAN SOCIETY OF ANIMAL SCIENCE

As a member of ASAS, we are sure you are aware of all the benefits that having a professional membership offer, including complimentary registration to monthly webinars and early access to the recordings, access to ASAS Journals, and discounted meeting registration to name a few. Remember to renew your ASAS membership or join ASAS to take advantage of these benefits.

More information on memberships can be found on the Membership website, https://www.asas.org/join-asas/individual-membership.

### 2024 ASAS Meetings

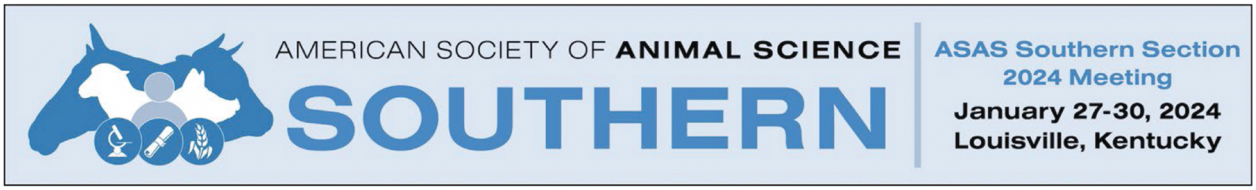

While Kentucky is known as the center for bourbon and horses, you won't want to miss this opportunity to round up the rest of the livestock at this year's **ASAS Southern Section meeting in Louisville, KY from January 27-30, 2024.**

The annual meeting will be held at the state-of-the-art Omni Hotel in downtown Louisville and offers great amenities for the event’s educational sessions and networking opportunities. Graduate and undergraduate students will have the opportunity to interact with industry leaders and brush up their skills on telling their animal science story to the public. Professional attendees can choose from multiple symposia and invited lectures on various livestock management topics. The Randel Lecture Series is a focal point for the meeting with Dr. Russ Anthony as the invited lecturer. Dr. Anthony is highly recognized within ASAS and was the recipient of the 2023 ASAS Physiology and Endocrinology Award. The SERA 41 and Kunkle Symposium will tackle issues surrounding beef cattle sustainability from both an applied and basic perspective. The SERA 41 symposia will focus on methodology for measuring feed intake and greenhouse gas emissions. The symposia will address methods, funding opportunities, and success stories of work in this area among the section. The Kunkle symposia will partner with Elanco to provide a perspective on improving sustainability in forage-based beef production systems, which includes idea sharing from industry and academia.

For more information about the meeting including schedule, registration, and housing. Please visit, https://www.asas.org/sections/southern-section/meetings.

AMERICAN SOCIETY OF **ANIMAL SCIENCE**
**MIDWEST**
**ASAS Midwest Section 2024 Meeting**
**March 10-13, 2024**
**Madison, Wisconsin**

**The 2024 ASAS Midwest Section Meeting** will be held in Madison, Wisconsin, March 10-13, 2024, at the Monona Terrace.

The program committee has been busy coordinating for a program that is rich in science for both our fundamental and translational colleagues! Symposia will include emerging topics across multiple disciplines including nutrition, growth, meat science, physiology, and teaching. We look forward to showcasing our undergraduate and graduate students in their competition papers. We also are excited for timely topic areas that are of interest to all members including effectively communicating animal science with consumers, animal agriculture and the climate, and how advances in technology can enhance production. The meeting is designed to bring academic, industry, and government scientists together to foster ideas for advancing animal agriculture.

Make sure to check the meeting website for more information about the meeting including housing and registration information.
https://www.asas.org/sections/midwest-section/meetings

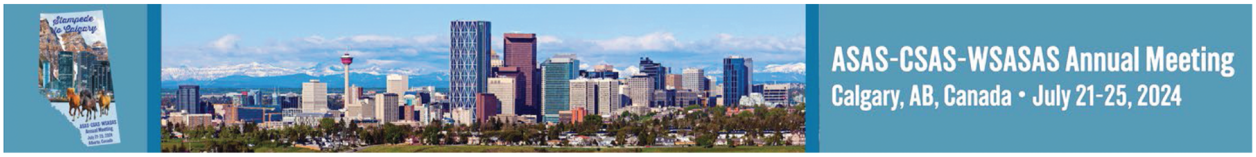

**The 2024 ASAS-CSAS-WSASAS Annual Meeting** will take place at the Calgary TELUS Convention Centre.

We are excited to be involved in development of symposia that are designed to encompass emerging fundamental and applied science from multiple disciplines including nutrition, genetics, reproduction, growth, meat science, behavior, and physiology as they apply across diverse species groups. The meeting sessions are intended to bring together scientists from industry, academia, and government and provide an opportunity for diverse ideas to inter-mingle for the greater good of animal agriculture and its relevance to human wellness. The program will feature a significant number of graduate student presentations in addition to invited talks, providing opportunity for networking among emerging student scientists and academic and industry professionals.

In addition to the annual meeting, there are pre-meeting symposia scheduled such as the Biannual Reproduction Symposium and the Phileo Symposium on Pre-, Pro-, and Post-biotics.

Make sure to check the meeting website for registration and housing information, https://www.asas.org/meetings/annual-2024.

### EAAP
### European Federation of Animal Science

**THE 76th EAAP ANNUAL MEETING WILL BE HELD IN INNSBRUK (AUSTRIA) FROM 25th TO 29th AUGUST 2025**

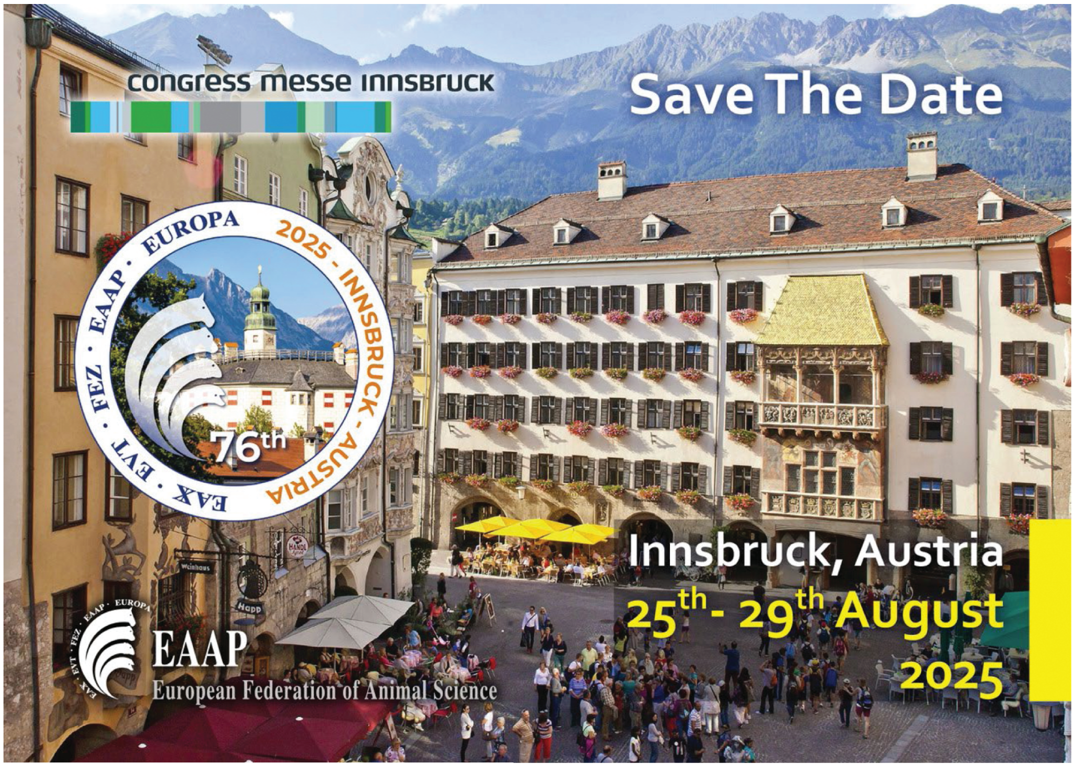

On behalf of the Austrian Organising Committee, we are very delighted to invite you to attend the 76th EAAP Annual Meeting 2024 to be held in Innsbruck (Austria), from 25th to 29th August 2025.
The sessions will be organized and jointly organized between the eleven EAAP Study Commissions to cover various areas of knowledge related to animal science, such as genetics, nutrition, physiology, animal health and welfare, precision livestock farming, horses, pigs, insects, livestock farming systems, cattle, sheep and goat. **Congress Website:** http:// www.eaap2025.org

Please follow us on Facebook and Twitter
https://www.facebook.com/EAAP.ORG
https://twitter.com/eaapofficial

INDIVIDUAL MEMBERSHIP OF EAAP

The EAAP membership is open to all scientists. It is a great opportunity to be update on the latest publications and other relevant information in the animal sector.
We have more than **6200 members** already!
Become a member of the most exciting international animal science network, joining us at
http://www.eaap.org/Content/Individual_Member_Information.html

THE EAAP NEWSLETTER FOR INDIVIDUAL MEMBERS IS NOW AVAILABLE ALSO IN CROATIAN, FRENCH, GERMAN, PORTUGUESE, ROMANIAN, SLOVENIAN, SPANISH, SLOVAK, ITALIAN AND POLISH!

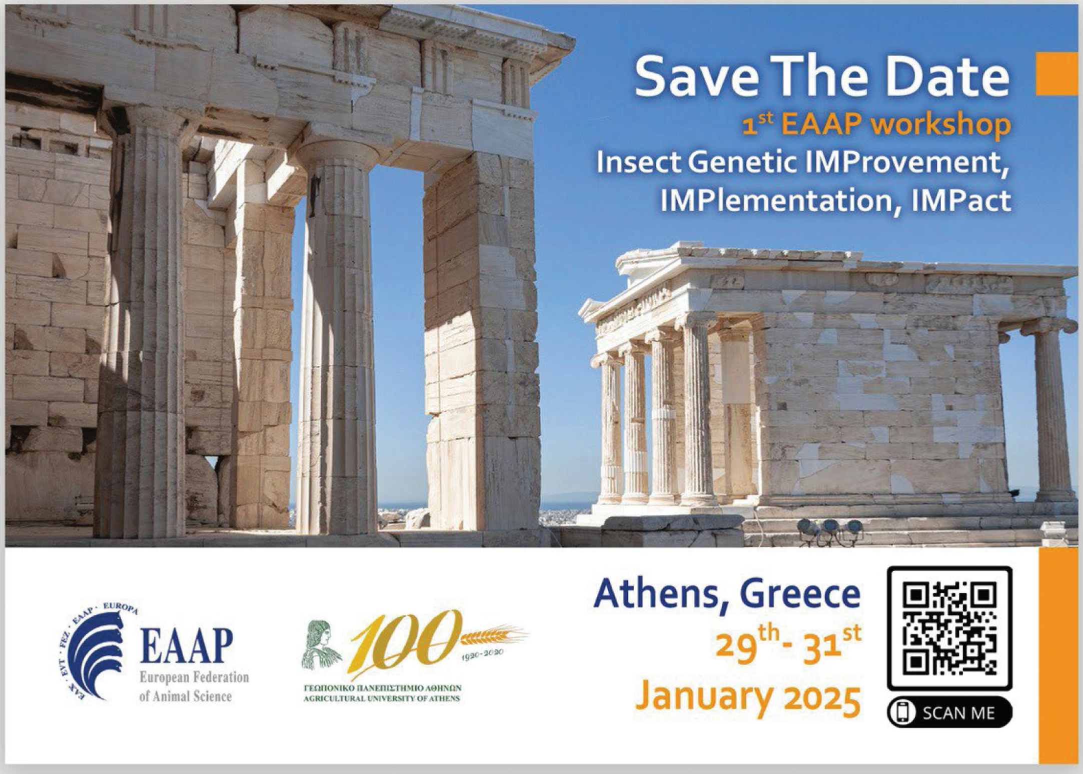

WORLD ASSOCIATION for ANIMAL PRODUCTION

The World Federation of Animal Science Societies and Associations, **WAAP**, is an International Non-Governmental Organisation with special consultative status with FAO since 1974.
The WAAP was established in 1965 after the success of the 1st World Conference on Animal Production held in Rome in 1963 organised by EAAP under the patronage of FAO.
WAAP aims to encourage closer collaboration between the animal production organisations and to bring together scientists, educators, technicians and administrators with the objective of reviewing development concerns on the five continents.

The Melbourne city at dusk

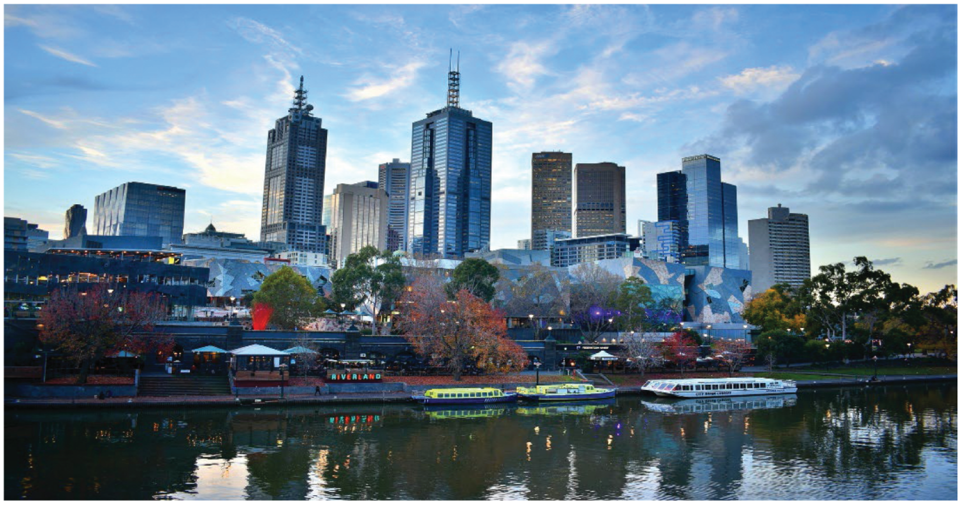

WAAP organises every five years a World Conference on Animal Production. Co-organized by EAAP and WAAP, the 2023 conference on “Climate change, biodiversity and global sustainability of animal production”, has been held in Lyon end August. It received 1900 abstracts among which 1200 were seleted for oral presentations in 96 sessions. More than 2200 delegates came to the conference. Ten sessions, organized or co-organized by WAAP, were video recorded and will be soon available by streaming on the EAAP Youtube channel. Two of them were especially attractive on the “Control of GHG emissions in ruminant farming” and on “Biodiversity as a lever for sustainable animal production”.

During the conference, WAAP delivered senior and junior awards and 3 student grants.

Philippe Chemineau ended his mandate after 5 years, Franck Dunshea was elected as the new President for 5 years. He will be in charge of organizing the next WCAP which will be held in Melbourne, Australia, in 2028.
